# Intraventricular ganglioglioma with dissemination of cerebrospinal fluid

**DOI:** 10.1590/0100-3984.2016.0222

**Published:** 2018

**Authors:** Patricia Pitta de Abreu, Bernardo Carvalho Muniz, Nina Ventura, Emerson Gasparetto, Edson Marchiori

**Affiliations:** 1 Instituto Estadual do Cérebro Paulo Niemeyer, Rio de Janeiro, RJ, Brazil; 2 Universidade Federal do Rio de Janeiro (UFRJ), Rio de Janeiro, RJ, Brazil

**Figure 1:**
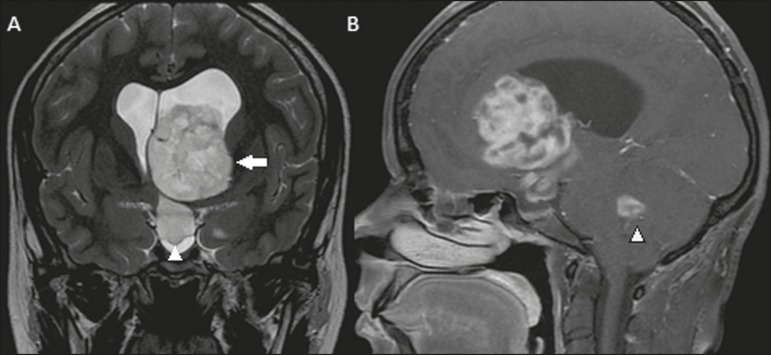
Coronal T2-weighted MRI sequence (**A**) and contrast-enhancedsagittal T1-weighted MRI sequence (**B**) showing a lesion with discretely elevated signal intensity on the T2-weighted sequence and heterogeneous contrast enhancement throughout the left lateral ventricle (arrow), extending through the foramen of Monro to the third ventricle (arrowhead in **A**). In the sagittal acquisition, the lesion can also be seen within the fourth ventricle (arrowhead in **B**).

**Figure 2:**
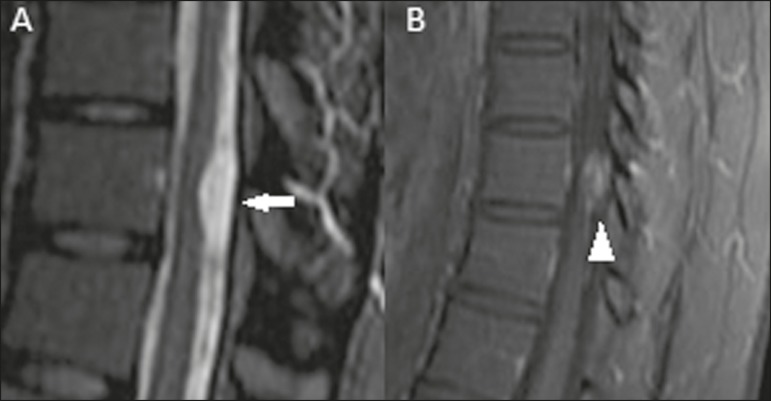
Sagittal T2-weighted MRI sequence (**A**) and contrast-enhanced sagittal T1-weighted MRI sequence (**B**) showing an intradural extramedullary lesion in the lower dorsal spine, with elevated signal intensity on the T2-weighted sequence (arrow) and intense contrast enhancement (arrowhead), consistent with leptomeningeal involvement.

Dear Editor,

A 26-year-old female patient presented with complaints of a bilateral reduction in visual acuity, headache, and generalized tonic-clonic seizures. Computed tomography of the brain revealed obstructive hydrocephalus, together with an expansile lesion occupying the third ventricle and extending to the left lateral ventricle. The presence of the intraventricular lesion was confirmed by magnetic resonance imaging (MRI), with signal intensity that was intermediate in a T1-weighted sequence and high in a T2-weighted sequence, showing contrast enhancement, as well as the invasion of the fourth ventricle (Figure 1). The patient was submitted to resection of the lesion that occupied the third ventricle. The tumor was drained, facilitating the clearing of the foramen of Monro and consequent resolution of the hydrocephalus. There was bilateral improvement of the visual turbidity, although the visual deficit persisted in the left eye, without other neurological deficits. The histopathological report described glial and neuronal neoplasia with ganglion cells, consistent with World Health Organization (WHO) grade I ganglioglioma. Subsequently, the patient underwent lumbar puncture with collection of cerebrospinal fluid (CSF), which was found to contain neoplastic cells. MRI of the lumbar spine revealed an intraspinal extramedullary lesion, with high signal intensity in a T2-weighted sequence and contrast enhancement, in contact with the posterolateral aspect of the spinal cord (Figure 2), suggestive of CSF dissemination.

A number of recent studies have emphasized the importance of MRI in assessing the central nervous system^1-3^, especially in relation to brain tumors^4,5^. Gangliogliomas are rare tumors, accounting for 0.33-1.3% of all primary brain tumors^6^. They mainly affect children and young adults. They are considered mixed tumors because they have neuronal and glial components. These tumors are typically of low grade (WHO grade I or II), with very low rate of malignancy. The most common location of a ganglioglioma is the temporal lobe, in which case the main symptom is refractory epilepsy, although it can occur at any location within the brain or even in an extraparenchymal location, as in the case of intraventricular gangliogliomas^6,7^. On MRI, gangliogliomas can present as cystic, solid-cystic, or completely solid lesions, typically with contrast uptake. However, the absence of enhancement does not exclude the diagnosis^8^. Intraventricular gangliogliomas are quite rare, few cases having been reported in the literature. The symptoms of intraventricular gangliogliomas differ from those of intraparenchymal gangliogliomas, the former typically not being associated with epilepsy. The symptoms of intraventricular ganglioglioma are caused by obstruction of CSF flow and hydrocephalus, headache and visual impairment being common^8^. According to various reports^6-11^, intraventricular gangliogliomas can originate in the lateral ventricles, in the third ventricle, and fourth ventricles-some even originating in the choroid plexus-and should always be included in the differential diagnosis of intraventricular lesions.

The case presented here was one of an intraventricular ganglioglioma apparently originating in the third ventricle, extending to the lateral ventricles and the fourth ventricle, the histopathological diagnosis being WHO grade I ganglioglioma with signs of CSF dissemination during subsequent examinations. In conclusion, a diagnosis of ganglioglioma should be considered in the presence of intraventricular lesions. In addition, imaging of the neuroaxis is recommended, regardless of the histopathological grade of the lesion, because CSF dissemination has been reported in the monitoring of other low-grade tumors, including gangliogliomas^12,13^.
